# Development of a Midinfrared Cavity Ring-Down Spectrometer for High-Precision Analysis of Carbon Dioxide Clumped Isotopic Compositions

**DOI:** 10.1021/acs.analchem.6c00022

**Published:** 2026-04-20

**Authors:** Ningwu Liu, Kota Tsuge, Ryohei Terabayashi, Osamu Abe, Ryu Uemura, Hideki Tomita

**Affiliations:** 1 Department of Applied Energy, 12965 Nagoya University, Nagoya 464-8602, Japan; 2 Graduate School of Environmental Studies, 12965 Nagoya University, Nagoya 464-8601, Japan

## Abstract

A midinfrared cavity ring-down (CRD) spectrometer was developed employing a 4.44 μm quantum cascade laser (QCL) for the sensitive detection of CO_2_ isotopologues, including ^12^C^16^O_2_ (44), ^13^C^16^O_2_ (45), ^12^C^16^O^18^O (46), and ^13^C^16^O^18^O (47). Instead of using an acousto-optic modulator (AOM), rapid QCL current switching was employed to initiate ring-down events, effectively reducing optical losses typically introduced by the AOM and simplifying the system design. The spectrometer incorporated a high-finesse (∼50,000) optical cavity, achieving a minimum detectable absorption coefficient (MDAC) of 2.2 × 10^–11^ cm^–1^ with 14.5 s average time. A continuous frequency sweep within a 0.2 cm^–1^ spectral window enabled simultaneous analysis of the four target isotopologues. Allan deviation analysis over a 6 h continuous measurement revealed optimal detection precisions of 0.45 ‰ for δ^45^/^44^, 0.28 ‰ for δ^47^/^46^, 0.47 ‰ for δ^46^/^44^, 0.35 ‰ for δ^47^/^45^, and 0.3 ‰ for δ^47^/^44^ at 65 min averaging time, demonstrating excellent long-term stability and high detection precisions. Coupled with a dedicated gas handling system, the instrument enabled analysis of gas volumes as small as ∼10 mL. The CRDS system further demonstrated robust performance in real-sample analyses, exhibiting strong linear agreement with isotope ratio mass spectrometry (IRMS). High-precision determinations of the δ^13^C, δ^18^O, and δ ^47^/^44^ isotope ratios were successfully achieved.

## Graphical abstract

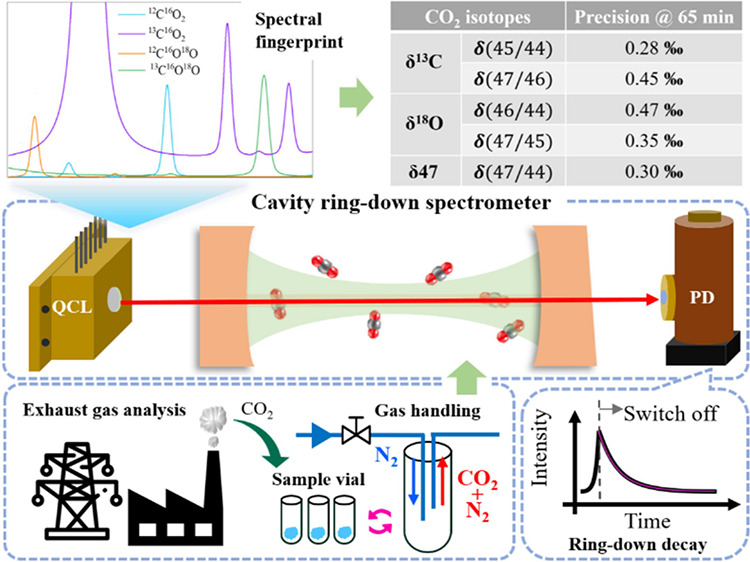

## Introduction

The analysis of stable carbon and oxygen isotopes in carbon dioxide offers valuable insights into environmental and climatic interactions^1–3^ and biomedical diagnostics.^4,5^ In environmental research, δ^13^C and δ^18^O measurements serve as indispensable tracers for clarifying complex atmospheric carbon cycling processes,^6,7^ enabling researchers to distinguish between natural and fossil fuel-derived CO_2_ emissions with unprecedented precision. These isotopic signatures provide critical constraints for quantifying carbon fluxes among terrestrial, oceanic, and atmospheric reservoirs while offering unique insights into ecosystem responses to climate change. Particularly, the distinct δ^13^C fingerprints of biogenic respiration versus fossil fuel combustion allow for robust source tracing in global carbon budget studies.^8^ Such differentiation is crucial for developing effective climate mitigation strategies and verifying the efficacy of carbon sequestration initiatives. Moreover, the determination of the abundance of the multiply substituted isotopologue ^13^C^18^O^16^O (Δ_47_) can be applied to paleoclimate temperature reconstructions,^9^ since variations in Δ_47_ in atmospheric CO_2_ are jointly determined by the source temperature and isotopic fractionation associated with diffusion processes. In clinical medicine, as established molecular biomarkers, changes in the contents of ^13^CO_2_, ^18^O^12^C^16^O, and ^12^CO_2_ in exhaled breath can be used to monitor multiple disease patterns, such as the diagnosis of *Helicobacter pylori* infections and noninvasive monitoring of glucose metabolism.^10,11^ In the ^13^C-urea breath test, the delta-overbaseline (DOB) value relative to standard references is used to indicate ^13^C and ^18^O enrichment, which enables the diagnosis of *Helicobacter pylori* infection based on its characteristic ability to hydrolyze urea and release isotopically labeled CO_2_. Therefore, long-term, accurate, and high-precision measurement of δ^13^C, δ^18^O, and Δ_47_ isotopes of CO_2_ is of great significance for the applications and promotion of research progress.

Gas-source isotope ratio mass spectrometry (IRMS)^12^ demonstrates a promising capability in terms of high precision and accuracy isotope detection. However, their routine establishment is limited by their huge instrumental dimensions. Additionally, due to the overlapping masses of some CO_2_ isotopologues and N_2_O molecules, IRMS is inevitably interfered by those gas components when measuring CO_2_ isotope samples. Moreover, the complex sample preparation and purification and high-power requirements make it unsuitable for real-time field applications. As an alternative, laser-based absorption spectroscopy^13–18^ has emerged as a powerful technique for isotope analysis, offering advantages such as high precision, fast response, and the potential for compact and field-deployable instrumentation. In addition, laser spectroscopy does not suffer from the mass overlap of multiple CO_2_ isotopologues because each CO_2_ isotopologue has specific absorption peaks. Midinfrared (MIR) absorption spectroscopy allows highly sensitive isotopic measurements to benefit from the characteristic of fundamental rotational–vibrational absorption bands of CO_2_ molecules. By exploiting this absorption regions, several laser absorption spectroscopy-based systems for clumped CO_2_ isotope analysis have been reported in recent years.^19–29^ Among these approaches, tunable diode laser absorption spectroscopy (TDLAS) has been most widely adopted owing to its relatively simple system configuration and flexible implementation.^25,26,28^ When combined with a multipass absorption cell, TDLAS enables high-sensitivity measurements of isotope ratios including ^12^C^18^O^16^O /^12^C^16^O_2_, ^13^C^16^O_2_/^12^C^16^O_2_, and ^12^C^17^O^16^O/^12^C^16^O_2_ isotopes.^19^ To further enhance detection performance, Wieman et al.^27^ introduced wavelength modulation spectroscopy (WMS) to suppress 1/*f* noise, significantly improving the signal-to-noise ratio (SNR). Subsequently, Wang et al.^29^ demonstrated clumped isotopic analysis of carbon dioxide via tunable infrared laser differential spectroscopy (TILDS), achieving a remarkable precision of 0.01‰ with 20 min of integration, thereby highlighting the great potential for clumped isotope studies. Meanwhile, to address the demands for reduced sample consumption and system miniaturization, hollow waveguide (HWG)-based spectrometers^30,31^ have been integrated into TDLAS systems, offering a viable pathway toward high-sensitivity isotope measurements with low gas consumption. However, the limited effective optical path length of the MPCs and HWGs necessitates the use of relatively strong absorption lines, typically exceeding 1 × 10^–23^ cm/molecule. This constraint makes it challenging to simultaneously measure the ^12^C^16^O_2_, ^13^C^16^O_2_, ^12^C^16^O^18^O, and ^13^C^16^O^18^O isotopologues using a single DFB-QCL. As a result, a dual-laser configuration was adopted in ref 29, targeting absorption features near 2250 and 2285 cm^–1^, which inevitably increased the system complexity and cost.

We address these limitations by employing cavity ring-down spectroscopy (CRDS), which has long been regarded as a benchmark for high precision trace-gas analysis. By measuring the decay time of light within a high-finesse optical cavity, CRDS enables detection of extremely weak absorption signals with minimal influence from fluctuations in the light source intensity.^9,32^ However, near-infrared CRDS systems^33–35^ have intrinsic limitations as only overtone absorption transitions of CO_2_ are accessible. The corresponding line strengths are rather too weak, particularly for multiply substituted isotopologues such as ^13^C^16^O^18^O. In contrast, when combined with midinfrared (MIR) laser sources, CRDS spectrometers can achieve substantially higher detection sensitivity due to the stronger fundamental vibrational–rotational transitions. Despite several studies involving radiocarbon isotope detection,^36–38^ MIR CRDS systems specifically dedicated to clumped CO_2_ isotopologue analysis have not yet been reported to the best of our knowledge.

In this work, we develop a first prototype of MIR cavity ring-down spectrometer for simultaneous measurement of the four CO_2_ isotopologues ^12^C^16^O_2_, ^13^C^16^O_2_, ^12^C^16^O^18^O, and ^13^C^16^O^18^O. A DFB-QCL, with a wavelength of around 4.4 μm, is applied to access the fundamental vibrational–rotational transitions of CO_2_ isotopologues. The CRDS spectrometer offers effective optical path lengths of ∼1.9 km, enabling the detection of weaker absorption transitions, 10^–25^ cm/molecule, and thereby allowing a single laser to access the multiple isotopologue transitions. A rapid laser current switching method is employed to replace the acousto-optic modulator (AOM) for laser interruption, enabling fast and precise control of the laser injection into the optical cavity. The minimum detectable absorption coefficient (MDAC) of 2.2 × 10^–11^ cm^–1^ is obtained at a 14.5 s average time. The system performance is validated through isotopic measurements of diverse samples, exhibiting excellent linearity with IRMS results and demonstrating high precision, stability, and repeatability.

## System Configuration

According to line-by-line calculation using the HITRAN2024 database,^39^ a wavenumber range from 2250 to 2255 cm^–1^ includes numerous absorption lines, as illustrated in Figure 1a. Among the several candidates of target lines, we selected a spectral window from 2252.75 to 2252.95 cm^–1^, as shown in the simulated spectrum in Figure 1b. Within this range, multiple absorption features of ^12^C^16^O_2_, ^13^C^16^O_2_, ^12^C^16^O^18^O, and ^13^C^16^O^18^O can be simultaneously detected. Equality in absorption coefficients between different isotopologues helps the accurate determination of isotope ratios. Nonetheless, a prominent ^13^C^16^O_2_ absorption peak at 2252.78 cm^–1^ is also present, which will be masked during the fitting process. To reduce spectral interference and improve the resolution of individual isotopologue lines, a low pressure of ∼13 mbar was employed.

**Figure 1:**
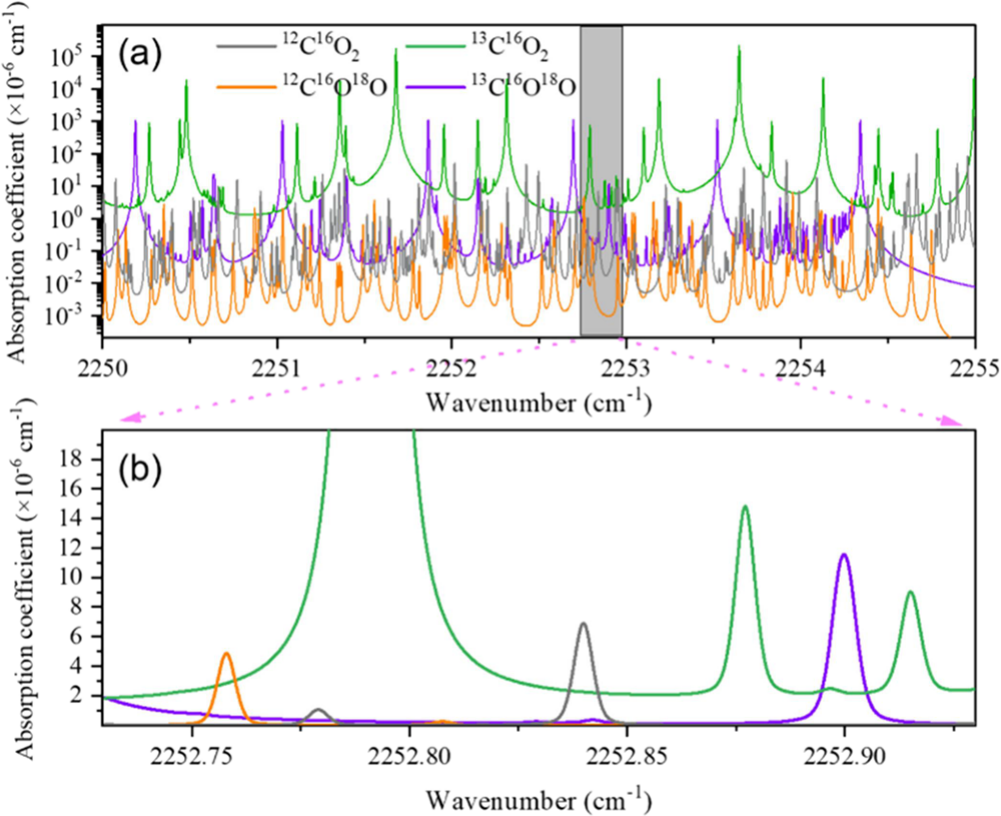
(a) Simulated absorption transitions of CO_2_ isotopes at the QCL emitted wavelength range and (b) simulated absorption coefficient of selected wavelength range 2252.75–2252.95 cm^–1^ with a CO_2_ concentration of 10%, pressure of 13 mbar, and temperature of 296 K.

The schematic of the CO_2_ isotope analysis system is depicted in Figure 2, mainly consisting of an optical spectrometer and a gas handling system. For the optical spectrometer, a 4.44 μm QCL (Hamamatsu Photonics, LE1855QCL) was employed to cover the spectrum range of 2250–2255 cm^–1^, shown in Figure 1a, and driven by a QCL driver (Hamamatsu Photonics, C16174–01). The laser beam was coupled to an optical isolator (Thorlabs, I4500W4) to prevent the QCL from back-reflected light, especially from a high-finesse cavity. After passing through a mode-match lens, the laser was introduced into the linear high-finesse optical cavity, whose length was 179 mm, yielding an FSR of 837 MHz. The cavity mirrors were mounted on a glass ceramic plate to ensure an extremely small thermal expansion. M1 and M2 in Figure 2 represent the ZnSe cavity mirrors, with a reflectivity of ∼99.9938% and a finesse of *F* = 5.0 × 10^4^. A piezoelectric actuator was implemented with M1, so that the cavity length could be slightly dithered to observe the optical resonance. After passing through the cavity, the cavity transmission is detected by a liquid-nitrogen-cooled InSb photodetector (Teledyne Judson Technologies, J10D-M204-R500U-30) with a transimpedance amplifier (FEMTO, HCA-1oM-100 K). The output signal of the photodetector is introduced to a comparator, which generates a TTL signal when the signal becomes above the threshold level. Then, a function generator sends a pulse signal into the current external modulation of the QCL driver to rapidly interrupt the resonance. Then, the ring-down signals are acquired and analyzed by a high-resolution digitizer (National Instruments, PXI-5922). To observe the absorption spectra, a sawtooth signal from the function generator, which is coupled into the fast-switching pulse, sweeps the laser current. The optical cavity was installed in a thermal insulation enclosure whose temperature was controlled by a heater (Thorlabs, HT10K).

**Figure 2:**
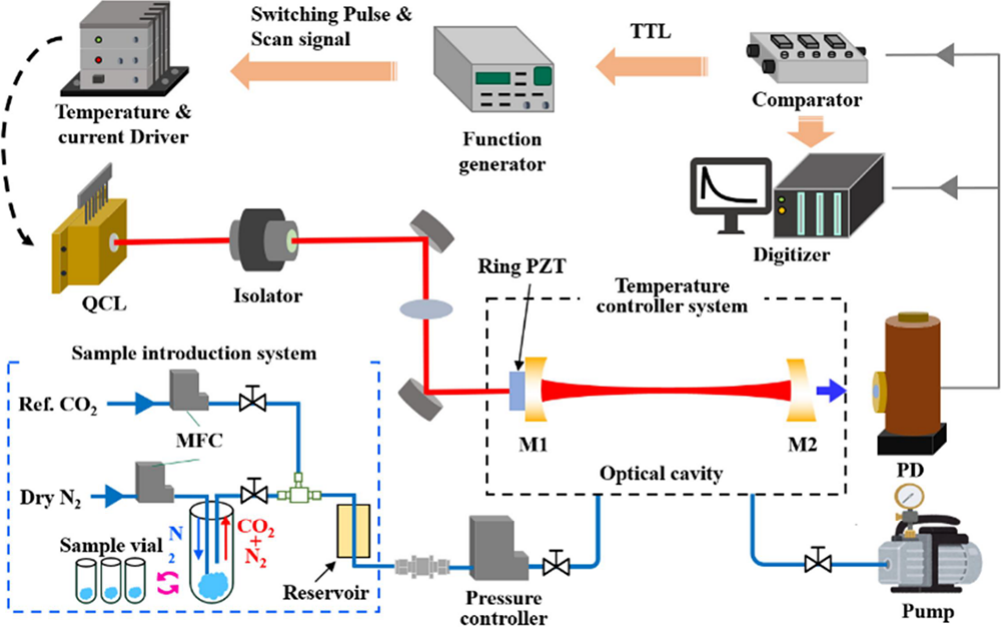
Schematic diagram of the MIR QCL-based cavity ring-down spectroscopic CO_2_ isotope analysis system, M: Mirror, PD: Photodetector, MFC: Mass flow controller.

The gas handling system comprises a sample introduction system, a pressure controller, and a vacuum pump. The samples can be introduced into the spectrometer in two ways: continuous mode and carrier gas mode. In continuous mode, the samples are connected to a needle valve directly, and the gas was continuously injected into the cavity, which is suitable for long-term measurement. However, the gas consumption will increase significantly according to the measurement time, yielding a flow of 0.3 L/h. In the carrier gas mode, the samples are prepared in a sample vial and then carried out using pure N_2_ gas flow. A mass flow controller (STECH INC, SEC-E40) is applied to control the gas flow of the carrier N_2_ gas. In this case, the gas consumption in each measurement is smaller, with a volume of ∼10 mL. However, the concentration of CO_2_ is diluted, which is suitable for a relatively high concentration of CO_2_ samples, generally more than 30%, to ensure a promising strong absorbance and high signal-to-noise ratio (SNR). A particle filter and a Teflon-based water filter are applied to filter out the dust and water vapor from the samples. The pressure in the optical cavity was maintained with a pressure controller (ALICAT, PCD-Series). A vacuum pump is used to provide the pressure difference for the gas flow.

## Results and Discussion

### System Performance

A rapid QCL current switching scheme is applied to interrupt the light buildup in the cavity without using an AOM, thereby reducing the system complexity and cost and avoiding optical power loss associated with AOM. In this scheme, the laser current is modulated by a sawtooth sweep signal, typically operating at a frequency below 1 Hz. Photons are stored in the cavity once the laser wavelength resonates with the cavity mode. When the transmitted light intensity exceeds a predetermined threshold, a TTL trigger signal is generated by a comparator. Upon receipt of the TTL trigger, the function generator produces a switching pulse signal. The superposition of the pulse signal onto the laser sweep induces a rapid jump in the laser frequency; therefore, the laser injection is interrupted. Figure 3a presents the time series of the signals: the blue line represents the TTL signal from the comparator, and the red line indicates the QCL switching pulse. The switching pulse has an amplitude of 160 mV, yielding a dithering wavelength of ∼0.0025 cm^–1^. Notably, a larger dithering wavelength is unfavorable for the temperature stability of the QCL. The duration of the pulse is 70 μs, ensuring that no further laser building occurs during the ring-down period. The observed ring-down decay signal is presented in Figure 3b.

**Figure 3:**
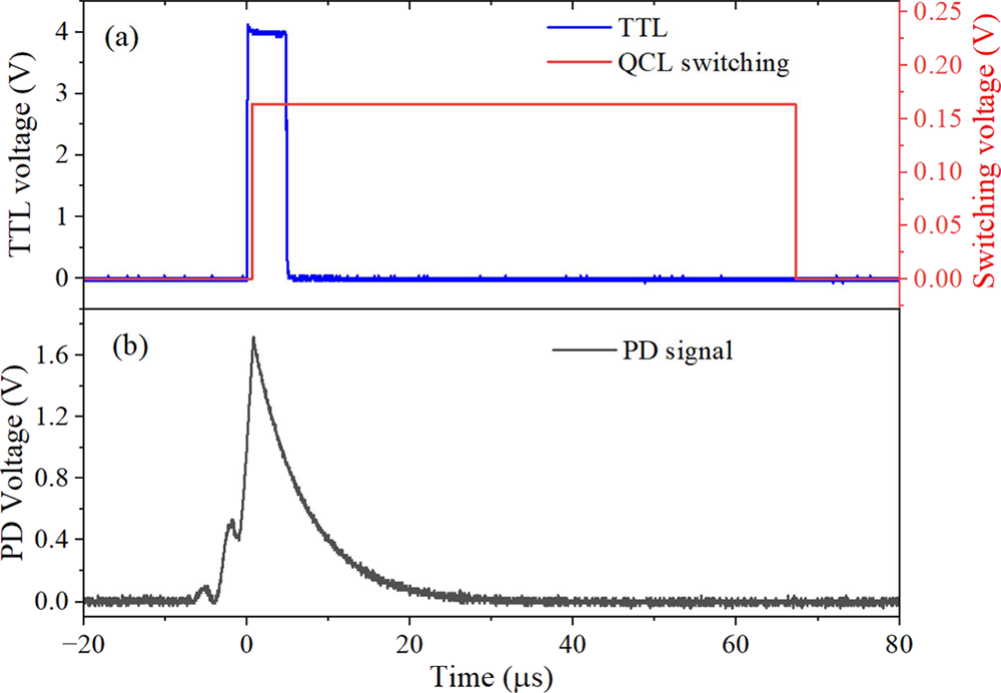
(a) Time series of the TTL signal (blue line) from the comparator and QCL switching pulse signal (red line) and (b) time series of the ring-down signal with the fast laser current modulation scheme.

Figure 4a shows the ring-down decay measured at a fixed wavelength of 2252.85 cm^–1^. The vertical axis shows the measured signal voltage corresponding to the light intensity, and the horizontal axis represents the decay time. Here, a least-squares exponential fitting is used to observe the ring-down time. The lower panel shows the fitting residual, with a standard deviation of 4.5 mV, indicating that the ring-down decay is well explained by an exponential function. The fitted ring-down time τ is 6.33 μs in pure N_2_, yielding an effective path length of ∼1.9 km. The ring-down signal was recorded with an acquisition rate *f* of 300 Hz and time of 100 s. The Allan–Werle deviation^40^ is plotted together with ideal Allan deviations for white noise versus integral time as presented in Figure 4b. The sensitivity of the spectrometer can be evaluated by the minimum detectable absorption coefficient (MDAC) or the noise equivalent absorption coefficient (NEAC).^41^ The MDAC obtained in a single shot is defined by α_min_ = σ­(τ)/(cτ^2^) and the NEAC = α_min_/ (*f*)^1/2^ $\sqrt{2/f}$ , respectively, where σ­(τ) is the standard deviation of τ and *f* is the repetition rate of measurement of τ. In this setup, we obtained a single-shot MDAC of 1.55 × 10^–9^ cm^–1^ and NEAC of 8.9 × 10^–11^ cm^–1^ Hz^–1/2^. The MDAC can be reduced by averaging 14.5 s evaluated to be 2 × 10^–11^ cm^–1^, demonstrating an extremely high detection sensitivity.

**Figure 4:**
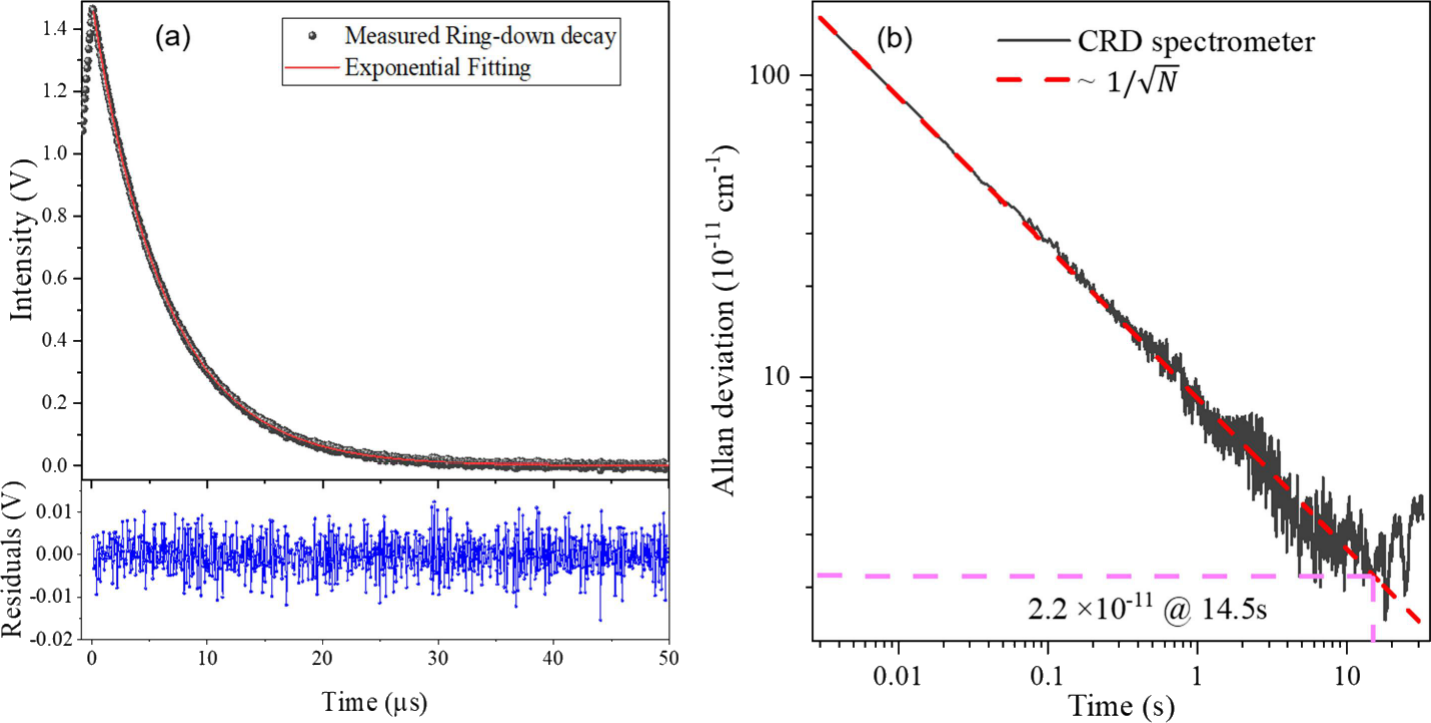
(a) Measured ring-down decay and exponential fitting (upper panel), together with the residuals of the exponential fitting (lower panel), (b) Allan–Werle deviations of the measured (cτ)^−1^ (black line) and ∼1/√*N* for comparison (red line), where *N* is the sample number of the ring-down signal.

Then, the CO_2_ spectra were measured using the CRD spectrometer. The QCL frequency was swept by the sawtooth 0.1 Hz sawtooth signal, with a PZT scanning frequency of 50 Hz and amplitude of 3.3 V. Figure 5 shows one of the measured spectra with the 5% CO_2_ mixed with N_2_ presented as black dots. The laser frequency was primarily calibrated according to the QCL current. However, the laser frequency has a nonlinear relationship with the modulation current; therefore, the absorption peak positions of the measured spectrum were used to further calibrate the actual laser frequency. The experimental pressure was 13 mbar, allowing the different absorption structures to be resolved. Nevertheless, some minor overlap remains—to address this issue, a multiple linear regression spectral fitting algorithm using the Voigt profile is adopted to separate and quantify the contributions from each isotopologues. The red solid line in Figure 5 reveals the all-sum of the fitted lines as well as the spectral components of each isotope (dashed line). Stable isotopic ratios are commonly expressed as ‘δ value’ in parts per million relative to the reference standard. For carbon and oxygen isotopes in CO_2_, the δ values are defined as follows:^42^

$$\delta =\left(\frac{R_{\mathrm{sample}}-R_{\mathrm{ref}}}{R_{\mathrm{ref}}}\right)\times 1000$$ 1

where *R*_sample_ and *R*_ref_ refer to the isotope ratio in the CO_2_ sample to be analyzed and reference ratio, respectively. In absorption spectroscopy, the isotope ratios (e.g., ^13^C/^12^C) can be determined with the ratio of the integrated area *A* and the absorption intensity *S* of the major and measured isotopic components:

$$R^{13}C=\frac{13A\times 13n\times 12S}{12A\times 12n\times 13S}$$ 2

where *n* is the isotope nature abundance and *n* and *S* are available from the high-resolution transmission molecular absorption database (HITRAN 2024).

**Figure 5:**
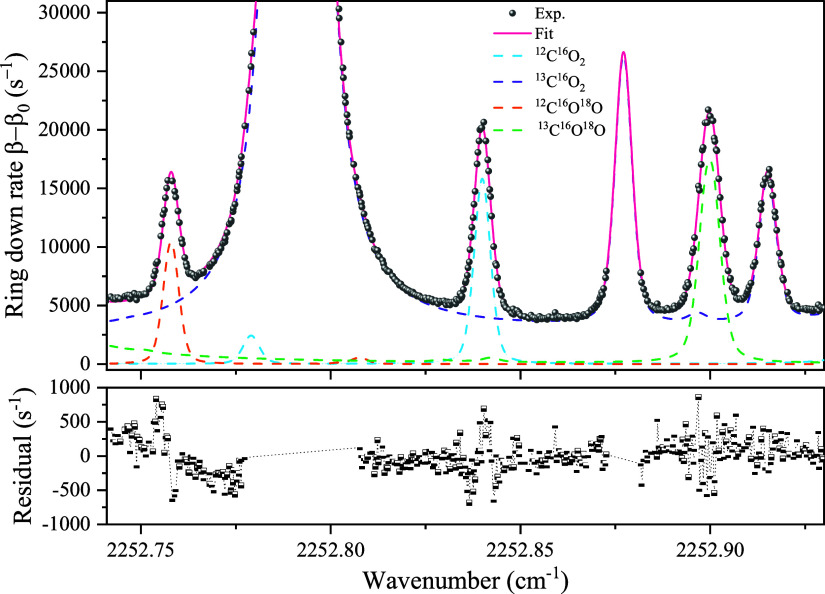
Measured spectrum with 5% CO_2_ concentration (black dot) and multiple linear regression spectral fitting (red line) based on the HITRAN 2024 database and fitted spectrum of different target isotopes (dashed line); the lower panel shows the residuals of the fitting.

The precision of the system for the isotope ratio analysis was evaluated by performing long-term measurements. A 5% standard CO_2_ gas was continuously introduced into the spectrometer with a flow rate of ∼5 standard ccm. The measurements were performed for over 6 h, with one spectrum acquired every 10 s. The measured isotopes ratios are shown in Figure 6, in which Figure 6a presents the measured R^13^C and Figure 6b presents the measured R^18^O and R^47^/^44^. The Allan deviation plots of R^13^C, R^18^O, and R^47^/^44^ are presented in Figure 6c–e, respectively. For R^13^C, short-term precisions of 11 and 9 ‰ are obtained for R^47^/^46^ and R^45^/^44^, respectively, and the Allan deviations achieve their minima at the integration time of 65 min, corresponding to the precisions of 0.45 and 0.28 ‰. For R^18^O, the system presents precisions of 12 and 8 ‰ at 10 s average time for R^46^/^44^ and R^47^/^45^ and can be improved to 0.47 and 0.35 ‰ at 65 min average times, respectively. The precision of the measured R^47^/^44^ achieves 8 ‰ at an average time of 10 s and is improved to 0.30 ‰ at 65 min. The Allan deviation analysis results demonstrate excellent performance of the spectrometer in terms of precision and long-term stability.

**Figure 6:**
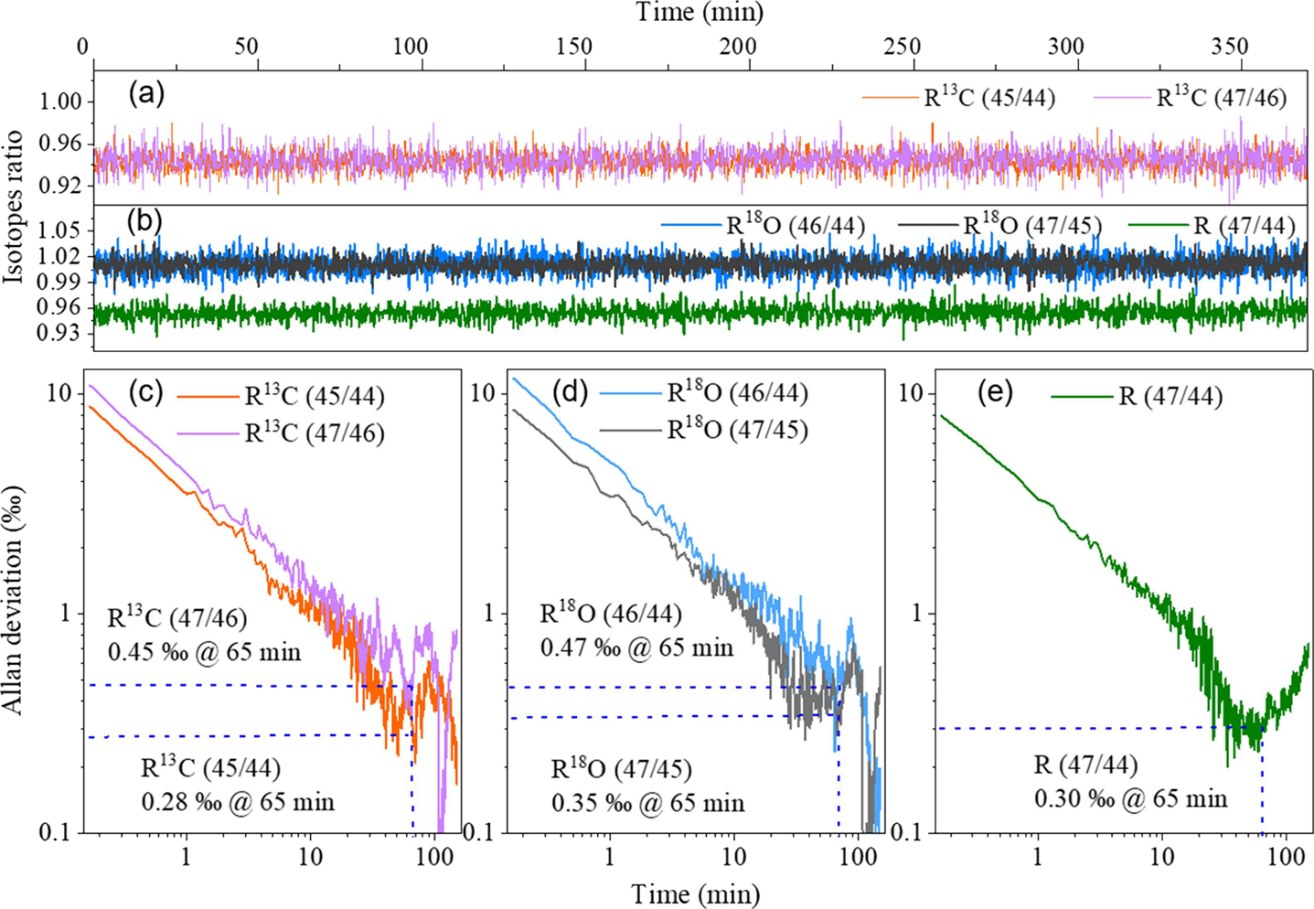
(a, b) Measured isotopes ratio of R^13^C, R^18^O, and R (47/44) with 11% of CO_2_ and (c–e) Allan deviation of the respective isotopic ratios as a function of measurement time.

### Isotope Detection with Small CO_2_ Volumes

The carrier gas scheme is used in small volume sample detection. Here, we used ∼35% CO_2_ standard gas samples and collected them in the 10 mL vials. By introduction of pure nitrogen, the CO_2_ gas can be carried out to the optical cavity. Figure 7a shows the dynamic CO_2_ concentration changes measured by a CRD spectrometer. The concentration of CO_2_ increases rapidly at the beginning followed by a gradual decrease after a plateau with the maximum concentration of ∼4.8% at 6 min. Here, we selected spectral data obtained around the time of 4.5 to 15 min (grayed area) to evaluate the isotope abundance since too low concentration (bellow 1%) of CO_2_ would deteriorate the detection precision due to a lower signal-to-noise ratio. Figure 7b–d presents the measured abundance of different isotopes as well as the natural abundance (black line) from the HITRAN database. The measured abundance of ^13^C/^12^C from 46/44 and 47/45 and ^18^O/^16^O from 45/44 and 47/46 presents similar values, respectively, demonstrating consistency in the analysis of isotopes with different molecules. The averaged values of the measured abundance are 0.01127 ± 1.64 × 10^–4^ and 0.01135 ± 2.038 × 10^–4^ for R^46^/^44^ and R^147^/^45^, 0.00393 ± 6.76 × 10^–5^ and 0.004 ± 4.706 × 10^–5^ for R^45^/^44^ and R^18^O^47^/^46^, and 4.58 × 10^–5^ ± 8.8 × 10^–7^ for R^47^/^44^, compared to the nature abundance 0.01106, 0.00394, and 4.43 × 10^–5^ for R^13^C, R^18^O and R^47^/^44^, respectively.

**Figure 7:**
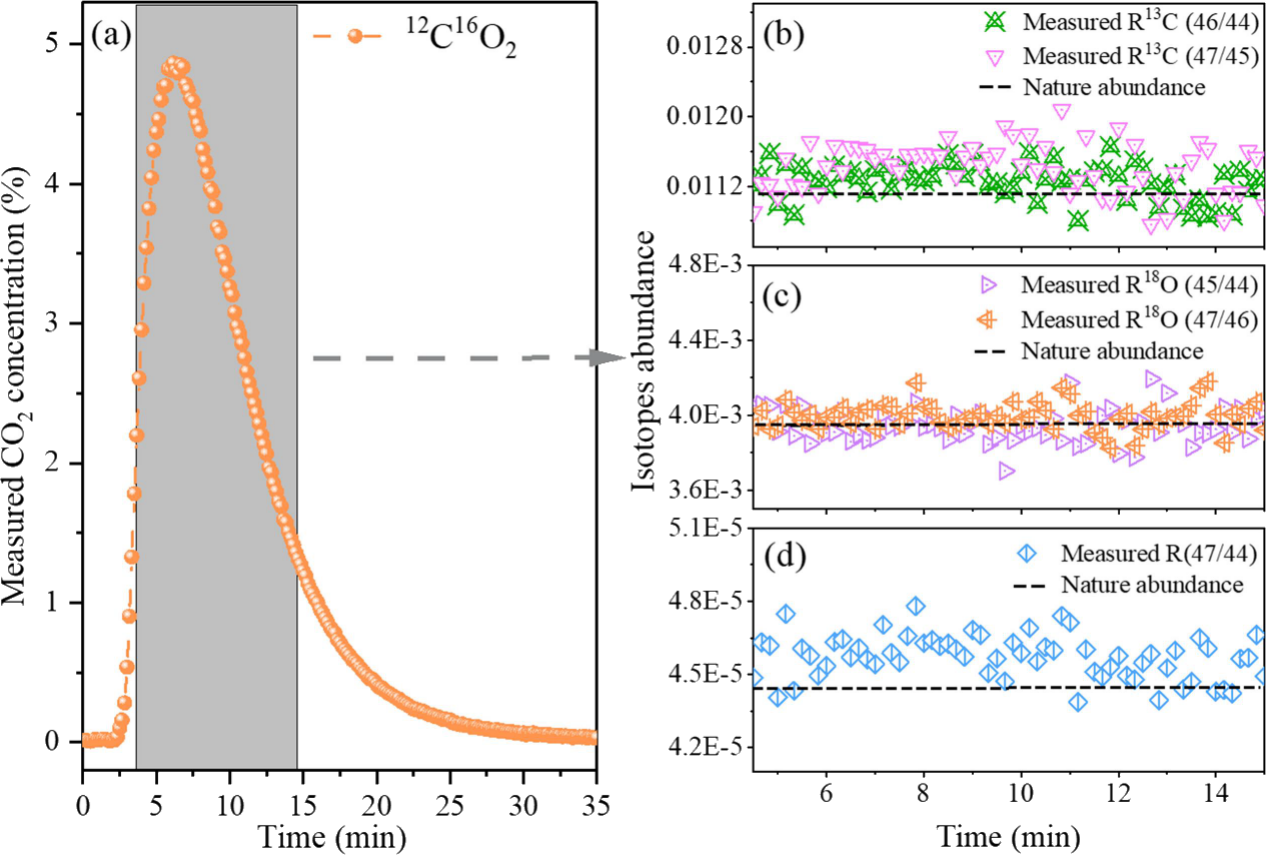
(a) CO_2_ concentration variations over the measurement time under the carrier gas handling scheme and (b–d) measured abundances of different isotopes, referenced to the natural abundances from the HITRAN database.

To evaluate the influence of the gas flow rate and scanning frequency of the QCL on the standard deviation (STD) of the measured isotope ratios, we varied the flow rate of the carrier N_2_ gas and the modulation frequency of the sawtooth wave independently. Figure 8a shows the STD of the isotope ratios as a function of the gas flow rates. In the experiments, we changed the flow rate of the pure N_2_ from 5 to 30 sccm and kept the laser scanning frequency of 0.1 Hz and pressure of 13.0 mbar. The experimental results show that the STDs were mainly increasing with the flow rate. Although R^13^C (45/44) exhibited a slightly different behavior, the overall trends indicated that a 5 sccm flow rate yielded a smaller STD value. Variations in the laser scanning frequency affect the acquisition time of each spectrum, thereby impacting the corresponding noise level. To clarify, different scanning frequencies, 0.1 Hz (10 s), 0.2 Hz (5 s), and 0.4 Hz (2.5 s), were applied, with the measured STD/√*f* presented in Figure 8b. The gas flow rate was kept as 5 sccm during the measurement. The STD/√*f* shows positive correlations with the scanning frequency of the QCL, and 0.1 Hz demonstrated the lowest STD values.

**Figure 8:**
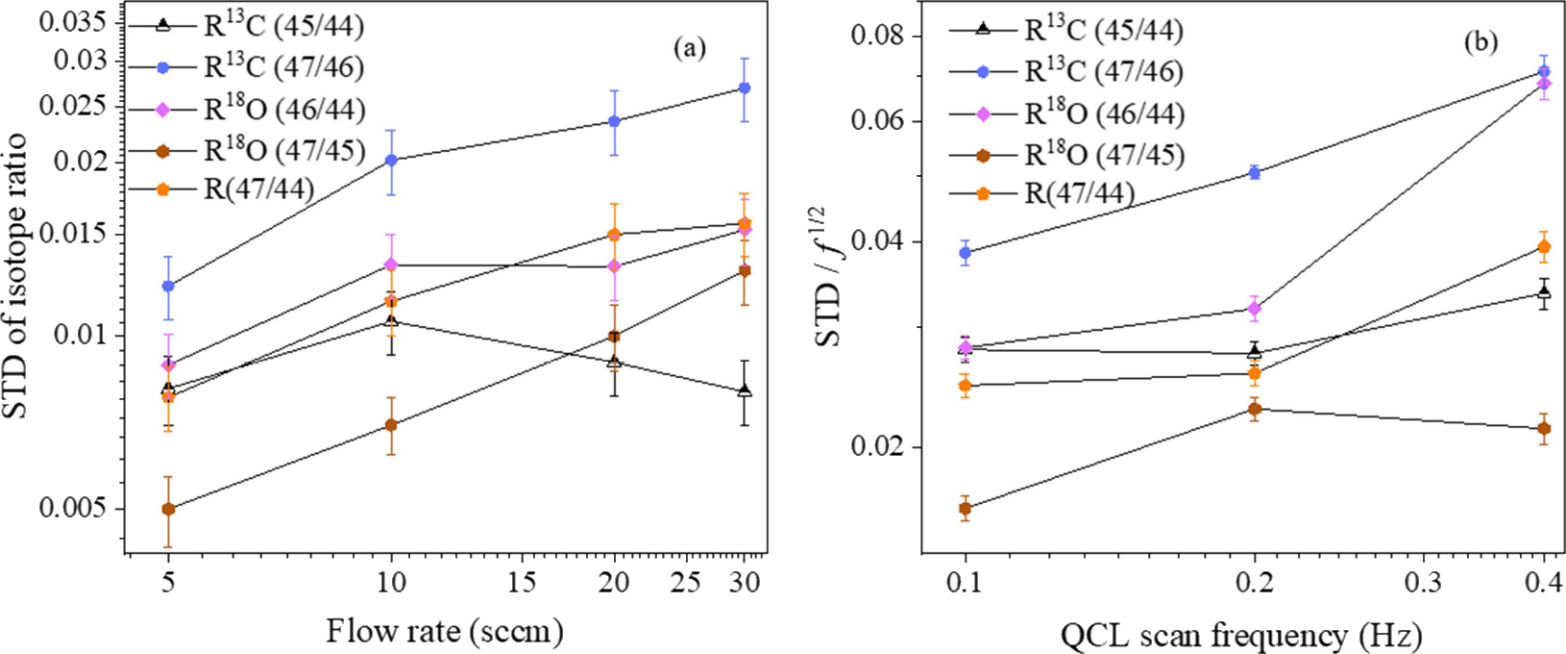
(a) The standard deviation (STD) of the measured isotope ratio for an individual measurement as a function of gas flow rate and (b) the dependence of STD/√*f* on the QCL scan frequency (*f*).

The performance of the CRD spectrometer operated under the carrier gas scheme was assessed by using five independent measurements of standard CO_2_ samples. Each measurement followed the procedure described previously. The δ values for each sample, along with the averaged values and standard errors (SE) of the resulting isotopic ratios, are summarized in Table 1. The averaged values for δ^45^/^44^, δ^47^/^46^, δ^46^/^44^, δ^47^/^45^, and δ^47^/^44^ were −48.51, −29.36, −7.60, 11.24, and −36.86 ‰, respectively. The corresponding SEs, calculated as SE = STD/√M (M = 5), were 0.28 0.83, 0.96, 0.33, and 0.28 ‰, respectively, demonstrating the excellent repeatability of the proposed spectrometer. The relatively larger SEs observed for δ^47^/^46^ and δ^46^/^44^ (0.83 and 0.96 ‰) are primarily attributed to spectral interference from the ^12^C^16^O^18^O isotopologue, which exhibits a stronger absorption line at 2252.78 cm^–1^. Although, the use of a multiple linear regression algorithm facilitates the identification of absorption peaks, spectral interference remains unavoidable, resulting in a slight reduction in sensitivity.

**Table 1:** Measured δ Values, and Standard Error of the ^13^C, ^18^O, and δ^47^/^44^ Isotopes from Separated Measurements

	δ^13^C (%_0_)		δ^18^O (%_0_)		δ47 (%_0_)
	δ (^45^/^44^)	δ (^47^/^46^)	δ (^46^/^44^)	δ (^47^/^46^)	δ (^47^/^44^)
#1	–48.54	–26.62	–10.20	11.03	–36.68
#2	–47.94	–29.85	–7.93	12.67	–37.72
#3	–48.60	–28.30	–8.63	11.13	–36.84
#4	–47.85	–32.25	–3.70	10.90	–35.86
#5	–49.62	–29.77	–7.54	10.47	–37.20
average (δ̅)	–48.51	–29.36	–7.60	11.24	–36.86
standard error	–0.28	0.83	0.96	0.33	–0.28

### Calcium Carbonate Sample Measurement

Following system optimization, calcium carbonate (CaCO_3_) samples were measured. Four CaCO_3_ samples, labeled as Sample 1 through Sample 4 (S1–S4) were prepared, with each having a mass of 10 mg. The samples were reacted with phosphoric acid (H_3_PO_4_) to generate CO_2_ gas and then subsequently collected in 10 mL vials. Four vials were prepared for each sample with a concentration of CO_2_ of ∼65%. For the δ^13^C determination, the isotopic ratios δ^45^/^44^ and δ^47^/^46^ were calculated separately, both exhibiting similar variations among the samples. Similarly, for δ^18^O, the δ^46^/^44^ and δ^47^/^45^ ratios displayed comparable trends, confirming the capability of the spectrometer for reliable δ^18^O determination. For validation, the same samples were analyzed using commercially available IRMS, and the results are summarized in Table 2. The δ^13^C values obtained via IRMS ranged from −1.1 to 1.1 ‰, while δ^18^O values showed a broader distribution from approximately −19 to 4.7 ‰. The δ^47^/^44^ ratio also exhibited significant variation, ranging from −1.1 to 23.2 ‰. Figure 9 illustrates the correlation between CRDS and IRMS measurements, revealing strong linear relationships, particularly for δ^18^O and δ^47^/^44^, with *R* ^2^ values of 0.997 and 0.97, respectively. The correlation for δ^13^C was comparatively lower (*R* ^2^ = 0.73), which is attributed to the limited variation of δ^13^C among the tested samples. Additionally, isotopic ratios δ^47^/^46^ and δ^47^/^45^ were analyzed exclusively by using the CRDS system. Their correlations with δ ^13^C and δ ^18^O, determined by IRMS, are plotted in Figure 9a,b, red dots, and exhibited differences from δ^45^/^44^ and δ^46^/^44^, but the overall trends remained consistent. The comparison between CRDS and IRMS measurements shows a difference in the absolute values of the isotopic ratios. The regression slopes slightly differ from one, and nonzero intercepts are observed. These discrepancies are primarily attributed to the systematic difference. Typically, uncertainties in spectroscopic line intensities and pressure-broadening parameters, especially for multiply substituted isotopologues, may introduce deviations. Moreover, the CRDS system determines molecular ratios, whereas the IRMS systems measure atomic ratios, which may inherently lead to differences between the values produced by the two techniques. Such systematic discrepancies are commonly reported in cross-platform isotope comparisons^43,44^ and do not compromise the demonstrated precision, linearity, or stability of the present system.

**Table 2:** Measured δ Values of ^13^C, ^18^O, and δ^47^/^44^ Isotopes from Different Samples Using Commercially Available IRMS

	δ^13^C (%_0_)	δ^18^O (%_0_)	δ47 (%_0_)
S1	0.90	–4.73	13.66
S2	–1.10	–11.42	4.72
S2	0.82	–18.95	–1.05
S4	1.13	4.04	23.24

**Figure 9:**
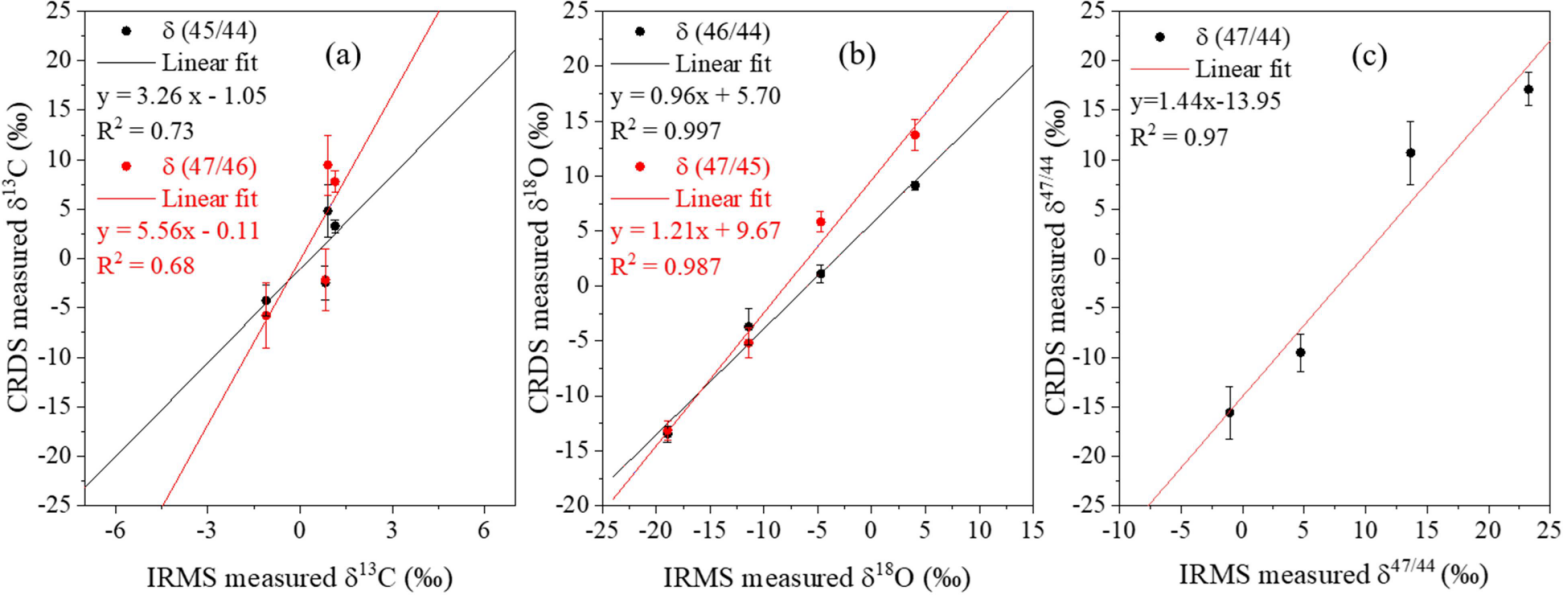
Measured (a) δ^13^C, (b) δ^18^O, and (c) δ^47^/^44^ values using cavity ring-down spectrometer as a function of the corresponding IRMS measurement results.

A comprehensive uncertainty assessment was performed. The statistical uncertainty (Type A uncertainty) was evaluated from the Allan deviation of repeated isotope retrievals. At an average time of 10 s, the Allan deviation is approximately 8 ‰ (R^47^/^44^). The corresponding fitting error of the absorption coefficient is 2.7 ‰, which propagates through the nonlinear isotope retrieval and results in the observed isotope deviation. With extended integration, the Allan deviation decreases to a minimum value of 0.3 ‰ at an averaging time of 65 min, demonstrating a low statistical uncertainty. The systematic uncertainty (Type B uncertainty) is primarily attributed to the spectroscopic parameters used in the retrieval algorithm. In particular, the line intensities of the selected transitions carry relative uncertainties of approximately 1–2% according to the HITRAN database.^39^ According to eq 2, uncertainties in the line intensity propagate as a multiplicative scale factor in the derived isotopic ratios. This effect explains the observed slope deviation from unity in the CRDS-IRMS fitting. It is worth noting that the selected transitions originate from the same absorption band and are modeled within a common spectroscopic framework. As a result, part of the line intensity uncertainty is correlated among isotopologues and partially cancels the isotope ratio determination. Additional systematic uncertainty arises from the stability of the gas temperature during the measurement. The temperature-related systematic deviation in the ^13^C quantification can be expressed as^45^

$$\Delta \delta \approx \frac{\Delta E}{kT^{2}}\Delta T\times 1000$$ 3

where *k* is the Boltzmann constant, *T* is the absolute temperature, and Δ*E* denotes the difference in lower-state energies of the selected transitions. Here, the temperature-dependent measurement uncertainty of Δδ^13^C is estimated to be 0.53 ‰, for *T* = 296 K and Δ*E* = 1609.5 cm^–1^ when the fluctuation of the gas temperature is controlled within 0.02 K.

The combined standard uncertainty is dominated by the spectroscopic parameter uncertainties, while the statistical component is below 0.3 ‰ at the optimal averaging time. Since the spectroscopic contribution acts as a multiplicative scale factor, it manifests as a calibration offset rather than random variation. This interpretation is supported by the high linearity observed in the CRDS–IRMS comparison. After calibration against IRMS, the systematic scale bias can be effectively removed, thereby improving absolute accuracy while preserving the intrinsic measurement precision of the CRDS system.

### Boiler Exhaust Gas Measurements

The CO_2_ isotopologues in exhaust gas samples from different boilers were measured using the developed CRDS spectrometer, denoted as Boiler 1 (power plant boiler) and Boiler 2 (industrial boiler), where Boiler 1 uses coal fuel and Boiler 2 uses liquefied natural gas (LNG) fuel. The exhaust gases were collected from the two boilers with four separate sampling bags for each. The measured δ values are presented in Figure 10, where #1–#4 correspond to the different sampling bags. The natural abundances from the HITRAN database are used as references. Figure 10a presents the measurements from Boiler 1. Relative to nature abundance, δ^13^C and δ ^47^/^44^ show negative values, whereas δ^18^O exhibits positive values. The averaged δ values are summarized in Figure 10b. A difference of 7.4 ‰ is observed between the δ^13^C values derived from δ^45^/^44^) and δ^47^/^46^. Similarly, the δ^18^O values calculated from δ^46^/^44^ and δ^47^/^45^ differ by 7.9 ‰. The discrepancies are likely attributable to the abundance of the ^13^C^18^O^16^O isotopologue. The results from the Boiler 2 samples are presented in Figure 10c,d. Similar discrepancies are also observed, with differences of 10.8 ‰ between δ^45^/^44^ and δ^47^/^46^, and 11.5 ‰ between δ^46^/^44^ and δ^47^/^45^, respectively. Moreover, the δ^13^C values obtained from Boiler 2 differ from those of Boiler 1 by 6.2 and 2.8 ‰ when derived from δ^45^/^44^ and δ^47^/^46^, respectively. The δ^18^O values also show differences between the two sources, with deviations of 10.8 and 7.2 ‰ for δ^46^/^44^ and δ^47^/^45^, respectively. These results indicate noticeable variations in the abundances of δ^13^C and δ^18^O-related isotopologues between the two combustion sources, demonstrating the strong capability of our MIR CRDS spectrometer for the field analysis of carbon dioxide clumped isotopes.

**Figure 10:**
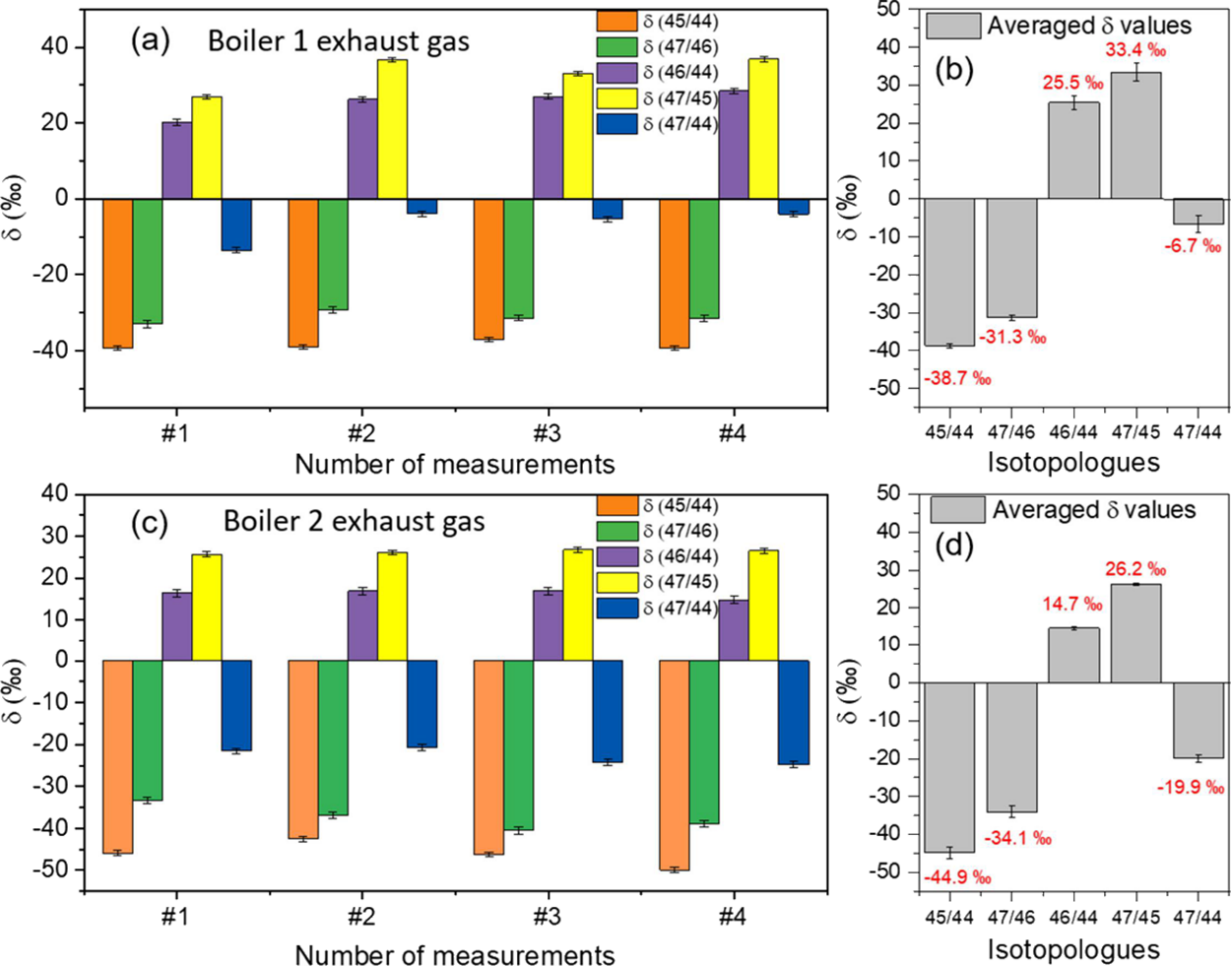
Measured isotopologue abundance from two boiler exhaust gas using the MIR cavity ring-down spectrometer.

## Conclusions

We developed an MIR CRDS system for high-sensitivity analysis of clumped CO_2_ isotopologues using a 4.44 μm QCL. The system incorporates a high-finesse optical cavity (∼50,000), providing an effective path length of 1.9 km. A rapid current-switching-based ring-down scheme was employed to reduce the optical losses and system complexity caused by the AOM. As a result, a minimum detectable absorption coefficient of 2.2 × 10^–11^ cm^–1^ was achieved at an integration time of 14.5 s. Despite targeting transitions with line strengths on the order of 10^–25^ cm/molecules, the spectrometer enabled precise measurement of multiple CO_2_ isotopic ratios. Simultaneous detection of four isotopologues, ^12^C^16^O_2_ (44), ^13^C^16^O_2_ (45), ^12^C^16^O^18^O (46), and ^13^C^16^O^18^O (47), was achieved within a narrow spectral window (2252.73–2252.96 cm^–1^). Allan deviation analysis over continuous 6 h measurements demonstrated isotope ratio precisions of better than 0.5 ‰, with optimal performance achieved at an integration time of approximately 65 min. The system was further validated through clumped isotope measurements of CO_2_ extracted from calcium carbonate samples using a carrier-gas-based gas handling system. The measured δ^13^C, δ^18^O, and δ^47^/^44^ values exhibited consistent trends and strong linear correlations with the IRMS results. In addition, measurements of boiler exhaust gases demonstrated good repeatability and robustness, highlighting the system’s suitability for field applications. Overall, these results demonstrate that the MIR CRDS system offers excellent sensitivity, stability, and quantitative accuracy for clumped CO_2_ isotopic analysis. This platform provides a promising tool for a wide range of isotope applications, including breath isotope analysis for metabolic studies and investigations of the atmospheric carbon cycle.
